# Plattenepithelkarzinom auf dem Boden eines oralen Lichen planus: Bericht zweier Fälle

**DOI:** 10.1007/s00105-020-04669-1

**Published:** 2020-08-20

**Authors:** Bijan Koushk-Jalali, Svenja Schürrle, Thomas Kuntz, Georgios Mitrakos, Christian Tigges, Frank Oellig, Andreas Hammacher, Steffi Silling, Ulrike Wieland, Alexander Kreuter

**Affiliations:** 1 Klinik für Dermatologie, Venerologie und Allergologie, HELIOS St. Elisabeth Klinik Oberhausen, Universität Witten/Herdecke, Josefstr. 3, 46045 Oberhausen, Deutschland; 2 Institut für Pathologie, Mülheim an der Ruhr, Deutschland; 3 Klinik für Mund‑, Kiefer- und Gesichtschirurgie, Malteser Krankenhaus St. Johannes-Stift, Duisburg, Deutschland; 4 Institut für Virologie, Nationales Referenzzentrum für Papillom- und Polyomaviren, Universität zu Köln, Köln, Deutschland

**Keywords:** Lichen planus mucosae, Oraler Lichen ruber, Fakultative Präkanzerose, Mukositis, Transformationsrate, Mucosal lichen planus, Oral lichen ruber, Premalignant condition, Mucositis, Transformation rate

## Abstract

Beim Lichen planus handelt es sich vermutlich um eine chronisch inflammatorische, immunologisch induzierte mukokutane Dermatose. Der Lichen planus mucosae manifestiert sich am häufigsten in der Mundhöhle. Diverse Triggerfaktoren wie bakterielle oder virale Infektionen, Medikamente oder physikalische Reize werden bei der Entstehung der Erkrankung diskutiert. Auch eine Assoziation mit Infektionen durch humane Papillomviren wurde beschrieben, ein kausaler Zusammenhang ist jedoch nicht hinreichend belegt. Als fakultative Präkanzerose kann sich auf dem Boden eines Lichen planus mucosae ein Plattenepithelkarzinom entwickeln, die maligne Transformationsrate ist aber gering. Das Risiko der malignen Transformation ist signifikant erhöht bei Patienten mit oralem Lichen planus, die rauchen, vermehrt Alkohol konsumieren oder an Hepatitis C erkrankt sind. Wir beschreiben 2 Patienten, bei denen sich lokal fortgeschrittene Plattenepithelkarzinome auf dem Boden eines langjährig bestehenden oralen Lichen planus entwickelten. Beide Fälle wurden erfolgreich durch radikale Tumorresektion mit anschließender Rekonstruktion und adjuvanter Radiatio/Radiochemotherapie behandelt.

## Abstract (en)

Lichen planus is a chronic inflammatory, immunologically mediated mucocutaneous dermatosis. Lichen planus mucosae predominantly affects the oral cavity. Various trigger factors such as bacterial or viral infections, drugs or physical stimuli are discussed in the development of the disease. An association with human papillomavirus infections has also been described, but is not sufficiently proven. Lichen planus mucosae is considered as a premalignant condition, but the malignant transformation rate is low. The risk of malignant transformation is significantly increased in patients with oral lichen planus who smoke, drink alcohol or have hepatitis C. We describe two patients with locally advanced squamous cell carcinoma that developed on a longstanding oral lichen planus. Both cases were successfully treated with radical tumor resection, subsequent tissue reconstruction, and adjuvant radiation/radiochemotherapy.

Kaum eine chronisch entzündliche Hauterkrankung hat ein so breites klinisches Spektrum wie der Lichen planus. Bei Beteiligung der Mundschleimhaut imponiert die Erkrankung oft als weißliche, nicht abwischbare Streifung (Wickham-Zeichnung), die im Verlauf auch zu schmerzhaften Erosionen und Ulzerationen führen kann. Die häufigsten oralen Lokalisationen sind Wangenschleimhaut, Zunge und Gingiva [1]. Chronisch aktive Verläufe eines Lichen planus mucosae können zur Entstehung von Karzinomvorläuferläsionen oder invasiven Plattenepithelkarzinomen führen. Demzufolge wird der orale Lichen planus von der Weltgesundheitsorganisation (WHO) auch als fakultative Präkanzerose oder „premalignant condition“ eingeordnet [2]. Wir berichten über 2 Fälle von lokal fortgeschrittenen Plattenepithelkarzinomen, die auf dem Boden eines langjährig bestehenden Lichen planus mucosae entstanden sind.

## Fallbericht 1

**Figure 1:**
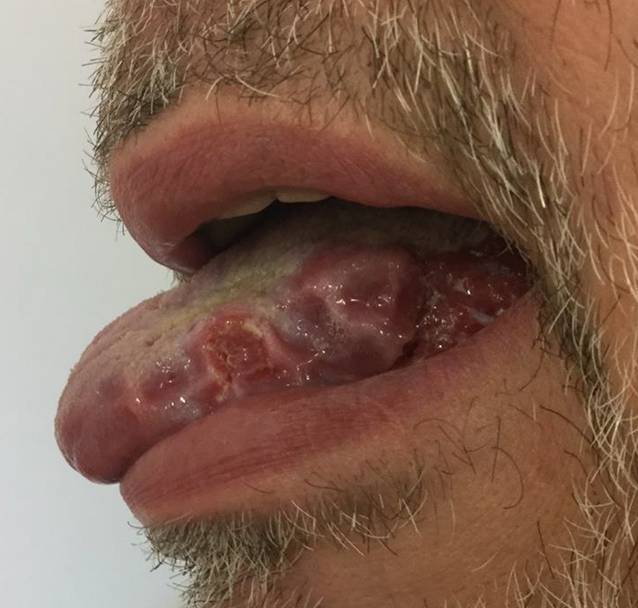

**Figure 2:**
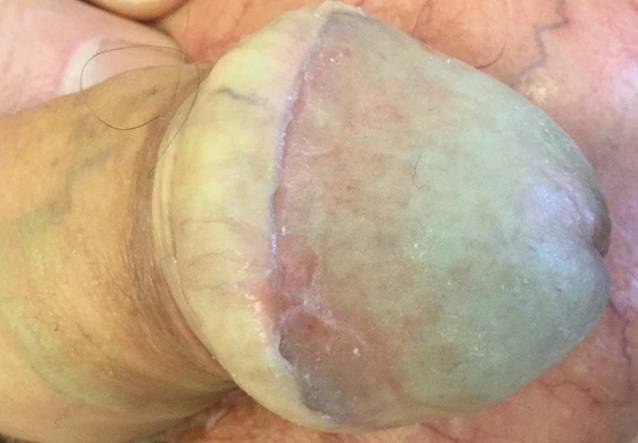

Ein 60-jähriger Patient stellte sich mit seit 3 Jahren bestehenden, langsam zunehmenden Schmerzen im Bereich der Zunge vor, die v. a. bei Nahrungs- und Flüssigkeitsaufnahme bestanden. Sieben Jahre zuvor sei anamnestisch ein Lichen planus der Mundschleimhaut diagnostiziert worden. Die bisher unregelmäßig erfolgte Lokaltherapie bestand aus der Applikation von Triamcinolonacetonid in Haftsalbe. Dies führte jedoch in den letzten Wochen zu keiner wesentlichen klinischen Besserung. Als Vorerkrankungen bestanden ein Diabetes mellitus Typ 2, arterielle Hypertonie und Vorhofflimmern. Eine aktive oder abgelaufene virale Hepatitis (Hepatitis A, B oder C) lag nicht vor. Übermäßiger Alkoholkonsum und Nikotinabusus wurden verneint. In der dermatologischen Untersuchung imponierten weißliche, zum Teil retikuläre, nicht abstreifbare Plaques an Wangenschleimhaut und Zunge. Zudem zeigten sich an der linken Zungenseite 2 umschriebene Ulzerationen (Abb. 1). Die weitere Inspektion der Haut zeigte keine auffälligen Befunde, im Genitalbereich fielen jedoch ebenfalls eine retikuläre, weißliche Streifung und außerdem Synechienbildung an Glans penis und Präputium mit beginnender Phimose auf (Abb. 2).

**Figure 3:**
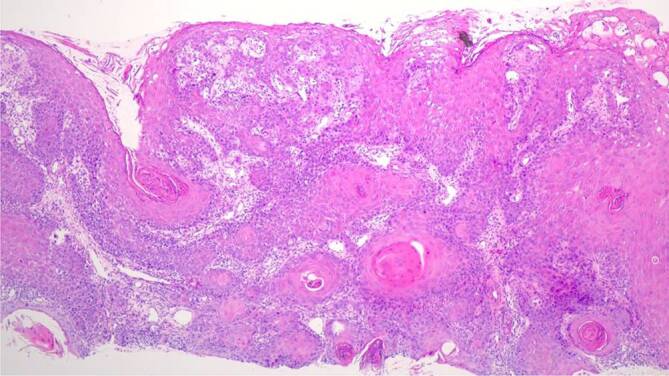

**Figure 4:**
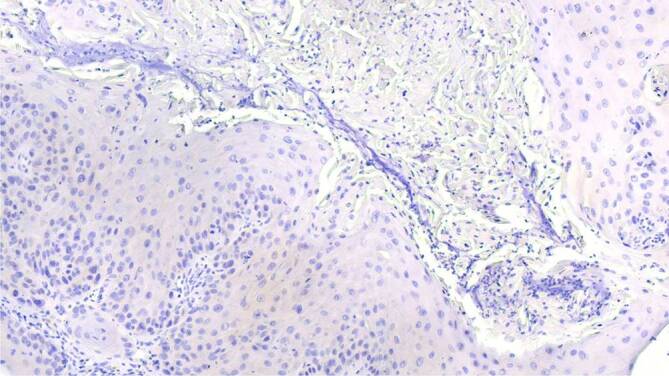

Zur weiterführenden histopathologischen Abklärung wurden tangentiale Exzisionen der beiden Ulzerationen am Zungenrand vorgenommen. In der histopathologischen Untersuchung zeigten sich 2 subtotal exzidierte, hochdifferenzierte, gering verhornende Plattenepithelkarzinome mit einer Tumordicke von 2,4 und 1,9 mm (Abb. 3). Die immunhistochemische Färbung für p16INK4a, einem indirekten Marker für Hochrisiko-HPV(humanes Papillomavirus)-Onkogen-Expression, fiel negativ aus (Abb. 4). Dazu passend war auch die virologische PCR(Polymerasekettenreaktion)‐Diagnostik für 38 HPV des Genus Alpha (Hoch- und Niedrigrisiko-HPV-Typen) negativ.

**Figure 5:**
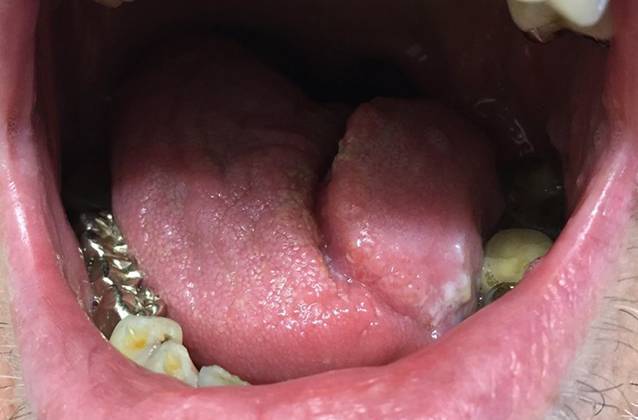

Zur weiteren operativen Sanierung der Zungenläsionen wurde der Patient in eine Klinik für Mund‑, Kiefer- und Gesichtschirurgie verlegt. Die präoperative Ausbreitungsdiagnostik inklusive Computertomographie von Hals und Thorax, Sonographie des Abdomens und Ösophagogastroduodenoskopie ergab keine pathologischen Befunde. Es erfolgte eine Zungenteilresektion links mit intraoperativer Schnellschnittdiagnostik, die nun ein 1,3 cm tief in die Muskulatur infiltrierend wachsendes Plattenepithelkarzinom mit Lymphangiosis carcinomatosa, aber ohne Gefäßeinbrüche oder perineurale Tumorausbreitung zeigte. Die Defektrekonstruktion erfolgte mittels eines mikrovaskulär reanastomosierten, perforatorgestielten, rein kutanen Hauttransplantates vom rechten Oberschenkel (Abb. 5). Zudem wurde eine Neck-Dissection durchgeführt, die keinen weiteren Tumorbefall zeigte. Unter Berücksichtigung der gesamten Befunde handelte es sich somit um ein Zungenkarzinom auf dem Boden eines Lichen planus mucosae mit der Tumorklassifikation pT3, G2, pN0, L1, V0, Pn0, R0. Nach kompletter Einheilung des Transplantates erfolgte eine adjuvante Radiochemotherapie (60 Gy-Strahlentherapie und kombinierte Chemotherapie mit Cisplatin und 5‑Fluoruracil); 24 Monate nach Radiochemotherapie war der Patient weiterhin tumorfrei. Aufgrund der durch den genitalen Befall des Lichen planus entstandenen Synechienbildung im Bereich des Penis erfolgte eine Zirkumzision, die komplikationslos verlief.

## Fallbericht 2

**Figure 6:**
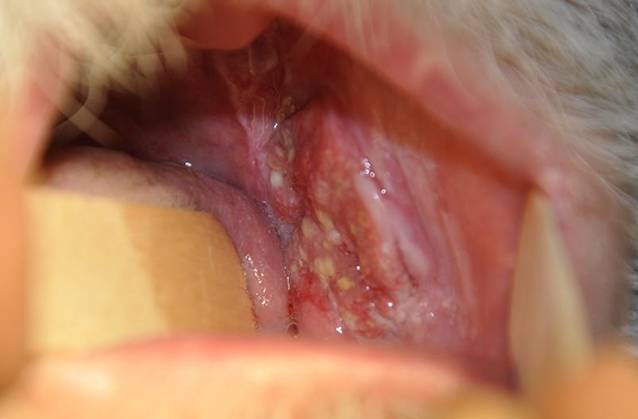

**Figure 7:**
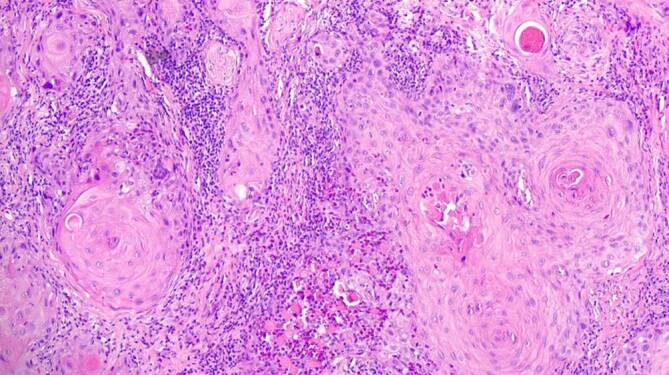

**Figure 8:**
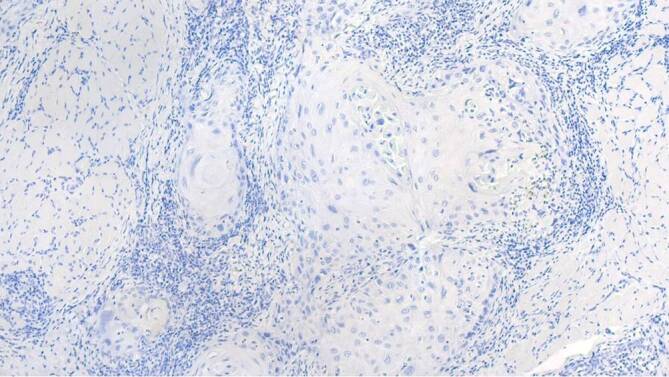

**Figure 9:**
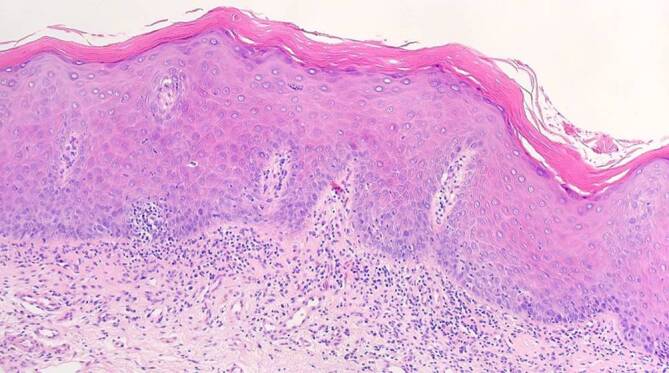

Ein 63-jähriger Patient stellte sich mit anamnestisch seit 4 Jahren bestehenden Schleimhautveränderungen bukkal links vor. Vor 1 Jahr sei nach Konsultation eines Zahnarztes eine Probebiopsie erfolgt, die das histopathologische Bild eines Lichen planus mucosae zeigte. Innerhalb der letzten 3 Monate sei es zu einer deutlichen Größenzunahme, vermehrten Schmerzen und Blutungen gekommen. Seit 3 Wochen bemerkte der Patient zudem eine Schwellung der Halslymphknoten. Eine Behandlung der Schleimhautläsionen war bisher nicht erfolgt. Als Vorerkrankungen bestanden eine arterielle Hypertonie sowie Zustand nach 2‑maligem Apoplex ohne bleibende neurologische Beeinträchtigungen. Eine Virushepatitis (Hepatitis A, B oder C) lag nicht vor. Alkoholkonsum bestand nicht, der Patient war jedoch starker Raucher (20 Packungs-Jahre). In der dermatologischen Untersuchung zeigte sich bukkal links ein tastbarer, derber, ca. 4 cm durchmessender Tumor mit zerklüfteter, zum Teil ulzerierter Oberfläche und weißlicher Streifung im Randbereich (Abb. 6). Zur histopathologischen Abklärung erfolgte eine Exzisionsbiopsie aus dem zentralen Anteil des Tumors. Hierbei zeigte sich ein hochdifferenziertes verhornendes Plattenepithelkarzinom mit bis in die Muskulatur reichenden Tumorausläufern (Abb. 7). Die immunhistochemische Färbung für p16INK4a sowie der HPV-DNA(Desoxyribonukleinsäure)-Nachweis mittels PCR (38 HPV-Typen) fielen negativ aus (Abb. 8). Eine Stanzbiopsie aus der weißlichen Streifung im Randbereich des Tumors zeigte das klassische Bild eines Lichen planus mucosae (Abb. 9). Zur weiterführenden Diagnostik erfolgte eine Magnetresonanztomographie des Halses. Hier fielen neben der Raumforderung der Wangenschleimhaut links 2 bis zu 18 mm durchmessende Lymphknoten links submandibulär auf. Die restliche Ausbreitungsdiagnostik war unauffällig. Zur weiteren operativen Sanierung erfolgte die Verlegung in eine Klinik für Mund‑, Kiefer- und Gesichtschirurgie. Hier wurde eine radikale Resektion des insgesamt 3 cm durchmessenden Tumors am Planum buccale links mit Unterkieferkastenresektion links, Osteotomie sowie Defektdeckung mittels Bichat-Lappen vorgenommen. Die komplettierende Neck-Dissection zeigte in 24 entnommenen Lymphknoten 2 Metastasen. Somit handelte es sich um ein Plattenepithelkarzinom der Mundhöhle auf dem Boden eines Lichen planus mucosae mit der Tumorklassifikation pT2, G2, pN1 (2/24), L0, V0, Pn0, R0. Es erfolgte eine adjuvante Radiatio mit einer Gesamtdosis von 60 Gy. Fünf Jahre nach abgeschlossener Radiatio ist der Patient weiterhin tumorfrei.

## Diskussion

Der Lichen planus ist eine chronisch inflammatorische, immunologisch induzierte mukokutane Dermatose, die vermutlich durch gegen basale Keratinozyten gerichtete CD8-positive T‑Zellen verursacht wird [3]. Diverse Triggerfaktoren wie bakterielle oder virale Infektionen, Medikamente oder physikalische Reize (z. B. Morsicatio, Zahnbehandlungen oder Zahnprothesen) werden bei der Entstehung des Lichen planus diskutiert. Auch Tabakrauch wird als irritierende Noxe diskutiert. Der Lichen planus mucosae manifestiert sich am häufigsten in der Mundhöhle. Nach Andreasen werden 6 verschiedene Formen unterschieden: retikulär, erosiv, papulös, plaqueförmig, bullös und atrophisch [4]. Die häufigsten Varianten sind dabei die retikuläre und die erosive Form [5]. Eine große retrospektive Studie mit 518 Patienten zeigte, dass der orale Lichen planus am häufigsten bukkal (87,8 %) und im Bereich der Zunge (56,9 %) auftritt [6]. Der Lichen planus mucosae kann sich jedoch auch im Larynx, im Ösophagus oder in der Genitalregion manifestieren. Der Anteil der an Lichen planus mucosae erkrankten Personen in der Bevölkerung wird auf ca. 0,5–2 % geschätzt, wobei Frauen doppelt so häufig betroffen sind wie Männer [7].

Der Lichen planus mucosae wird zu den fakultativen Präkanzerosen gezählt, die maligne Transformationsrate ist aber gering. In einer kürzlich publizierten Metaanalyse wurden insgesamt 12.838 Patienten mit oralem Lichen planus aus 33 Studien ausgewertet und eine Progressionsrate zum Plattenepithelkarzinom von 1,2 % ermittelt. Das Risiko der malignen Transformation ist bei Patienten mit oralem Lichen planus, die rauchen (Odds Ratio = 4,62), vermehrt Alkohol konsumieren (Odds Ratio = 3,22) oder eine chronische Hepatitis-C-Infektion haben (Odds Ratio = 3,77) signifikant erhöht [8].

Mundhöhlenkarzinome, wie in unseren beiden Fällen dargestellt, sind der häufigste Tumortyp unter den Kopf-Hals-Tumoren. Die geschätzte Anzahl an Neuerkrankungen liegt weltweit bei ca. 200.000 Fällen pro Jahr [9]. Mindestens ein Drittel aller oropharyngealen Karzinome ist HPV-induziert. Der Anteil der HPV-induzierten Mundhöhlenkarzinome ist mit 2,2 % deutlich geringer [9]. Die Assoziation von HPV mit oralem Lichen planus ist noch nicht abschließend geklärt. In einer Metaanalyse aus 22 Studien mit insgesamt 835 Patienten, die unter einem oralen Lichen planus litten, konnte eine HPV-Infektion in 35 % der Fälle (Odds Ratio = 6,83) nachgewiesen werden. Die Assoziation von HPV mit oralem Lichen planus scheint dabei starken geografischen Schwankungen zu unterliegen (Odds Ratio = 5,15 in Europa und Odds Ratio = 96,6 in Nordamerika) [10]. Interessanterweise scheint die HPV-Assoziation beim erosiven Lichen planus höher zu sein als bei nichterosiven Formen (Odds Ratio 9,34 vs. 4,32). Im Gegensatz dazu konnte in einer kürzlich publizierten Studie aus Spanien mit 83 prämalignen oralen Läsionen (41 davon oraler Lichen planus) nur in 4 Fällen (4,8 %) HPV-DNA nachgewiesen werden [11].

P16INK4a, ein indirekter Marker für HPV-Onkogen-Expression, wird in der überwiegenden Anzahl HPV-induzierter Dysplasien und Karzinome exprimiert. Zudem ist p16INK4a ein gut etablierter, validierter und prognostisch relevanter Biomarker bei Kopf-Hals-Tumoren [12]. In proliferierenden Geweben zahlreicher Tumorerkrankungen und auch bei chronisch entzündlichen Läsionen wird p16INK4a jedoch auch unabhängig von HPV exprimiert. So konnte auch beim oralen Lichen planus in einer Studie in mehr als 60 % der Fälle eine Überexpression von p16INK4a gezeigt werden [13]. In einer anderen Studie, in der 35 Fälle von oralem Lichen planus bezüglich p16INK4a und HPV-DNA untersucht wurden, konnte allerdings in lediglich 4 Läsionen HPV nachgewiesen werden [14].

Seit längerer Zeit ist bekannt, dass eine Infektion mit dem Hepatitis-C-Virus ein signifikant erhöhtes Risiko für die Entwicklung eines Lichen planus darstellt [15]. Durch die seit 2014 verfügbaren Kombinationstherapien, bestehend aus Protease‑, Polymerase- und NS5A-Hemmern, ist Hepatitis C heutzutage fast immer heilbar. Ein kürzlich veröffentlichter Review-Artikel hat unterschiedliche klinische Verläufe (81,8 % Abheilung/Besserung und 18,1 % Verschlechterung/Persistenz) beim Hepatitis-C-assoziierten Lichen planus nach erfolgreicher Hepatitistherapie gezeigt. Die Wahrscheinlichkeit einer kompletten Abheilung nach antiviraler Therapie war beim oralen Lichen planus am höchsten [16]. In den von uns präsentierten Fällen konnte jedoch eine Hepatitis als Triggerfaktor des oralen Lichen planus ausgeschlossen werden.

Alle kurativ resektablen Mundhöhlenkarzinome sollten, wenn es der Allgemeinzustand des Patienten zulässt, operativ behandelt werden. Bei fortgeschrittener T‑Kategorie (T3/T4), knappen oder positiven Resektionsrändern, perineuraler Invasion, Gefäßinvasion und/oder Lymphknotenbefall sollte laut AWMF (Arbeitsgemeinschaft der Wissenschaftlichen Medizinischen Fachgesellschaften e. V.) S3-Leitlinie eine postoperative Radio- oder Radiochemotherapie erfolgen [17]. Dieses Vorgehen war auch bei unseren Fällen erfolgreich. Bei ca. 20 % der Patienten kommt es allerdings zu einem Lokalrezidiv des Mundhöhlenkarzinoms, am häufigsten innerhalb der ersten 2 Jahre. Unsere beiden Patienten blieben im Nachbeobachtungszeitraum von 2 bzw. 5 Jahren tumorfrei.

Bei fortgeschrittenen, rezidivierten oder metastasierten Mundhöhlenkarzinomen wird bei Patienten in gutem Allgemeinzustand in der Palliativsituation eine Kombination aus platinhaltiger Chemotherapie (vorzugsweise Cisplatin) und 5‑Fluorouracil (Extreme-Schema) sowie dem „Epidermal-growth-factor-Rezeptor-Antikörper“ Cetuximab vorgeschlagen (https://www.awmf.org/leitlinien/detail/anmeldung/1/ll/007-100OL.html, Konsultationsfassung S3-Leitlinie Diagnostik und Therapie des Mundhöhlenkarzinoms). Für eine lange Zeit existierte kein Therapiestandard nach Progression unter bzw. nach der platinhaltigen Erstlinientherapie.

Seit Ende des Jahres 2016 besteht jedoch eine Zulassung der FDA (Food and Drug Administration) für den Checkpoint-Inhibitor Nivolumab bei Plattenepithelkarzinomen des Kopfes und Halses nach erfolgter platinbasierter Chemotherapie (Checkmate 141). Mitte des Jahres 2019 folgte die FDA-Zulassung von Pembrolizumab als Erstlinienbehandlung (Keynote 048) [18]. Obwohl unseres Wissens bisher noch nicht über den Einsatz von Checkpoint-Inhibitoren bei Plattenepithelkarzinomen auf dem Boden eines oralen Lichen planus berichtet wurde, sollte berücksichtigt werden, dass unter Nivolumab und Pembrolizumab auch Lichen-planus-artige Hautveränderungen beschrieben wurden und sich somit ein Lichen planus unter Checkpoint-Inhibition verschlechtern bzw. exazerbieren könnte [19].

Zusammenfassend verdeutlichen die hier dargestellten und ähnliche Fallberichte aus der Literatur, dass auf dem Boden eines chronisch aktiven mukosalen Lichen planus Plattenepithelkarzinome entstehen können [7, 20]. Obwohl das Risiko einer malignen Transformation insgesamt niedrig ist und in lediglich ca. 1 % aller Fälle auftritt, sollten bei Patienten mit mukosalem Lichen planus auch nach erfolgter Therapie regelmäßige klinische Kontrolluntersuchungen erfolgen.

## Fazit für die Praxis

- Beim chronisch aktiven oralen Lichen planus besteht ein geringes, aber dennoch erhöhtes Risiko für die Entstehung eines Plattenepithelkarzinoms.
- Die WHO (Weltgesundheitsorganisation) ordnet den oralen Lichen planus als fakultative Präkanzerose bzw. „premalignant condition“ ein.
- Eine Assoziation mit humanen Papillomviren ist beim oralen Lichen planus beschrieben, jedoch nicht gesichert.
- Patienten mit oralem Lichen planus sollten in regelmäßigen Abständen klinisch untersucht werden, um eine maligne Transformation frühestmöglich zu erkennen.
- Resektable Mundhöhlenkarzinome sollten primär operativ behandelt werden.
- Auch lokal fortgeschrittenere hochdifferenzierte Karzinome auf dem Boden eines oralen Lichen planus können kurativ behandelt werden.
